# Prediction factors for breast reconstruction postoperative complications

**Published:** 2013-12-25

**Authors:** MR Chiru, I Lascăr

**Affiliations:** Department of Plastic Surgery and Reconstructive Microsurgery, Clinical Emergency Hospital Bucharest

**Keywords:** breast reconstruction, complications

## Abstract

Abstract

Breast cancer is a major health problem that requires multiple forms of treatment, including surgery, adjuvant chemotherapy and radiotherapy and more recently, reconstructive surgery. The aim of this study is to determine the factors that can predict the chances of a patient having postoperative complications after breast reconstruction.

The surgical treatment for breast cancer combined with the new adjuvant therapies has significantly increased the overall survival in women with breast cancer, making it necessary to address this pathology not only from the oncologic point of view, but also in terms of the quality of life that these women will have. 

Since the primary treatment of breast cancer remains the surgery, the way to increase these patients’ well-being and quality of life and diminish the negative psychosocial effects of a mastectomy is to reconstruct the breast. 

When considering breast reconstruction there are two main things to take into account: the timing and the method of reconstruction. There are three options regarding the timing of post mastectomy breast reconstruction: 

• immediate reconstruction: mastectomy and reconstruction are performed at the same time. This procedure gives the best cosmetic result as it preserves the breast skin envelope and it has been proven a safe approach as well. In the event of a recurrence, the reconstructed breast does not impair the detection and treatment of the recurrent disease [**2**];

• delayed-immediate reconstruction is generally performed when the time of the mastectomy is uncertain if the patient requires or not postoperative radiotherapy. A tissue expander is inserted after the skin sparing mastectomy and the breast reconstruction will be performed at a later date; 

• delayed mastectomy involves two different surgical interventions, first the mastectomy and then the reconstruction after a period of at least as long as the time required for the patient to finish the radiotherapy and/or chemotherapy. 

 Regarding the method of breast reconstruction, there are also three approaches: 

• prostheses based reconstruction can be performed by the placement of an implant right after the mastectomy, or a tissular expander immediately after the mastectomy and the implant, at a later date. The ideal candidate for a breast reconstruction with an implant is a thin patient with bilateral reconstruction or a thin patient with one side reconstruction for a small, non ptotic breast [**1**]; 

• reconstruction with autologous tissue – pedicle or free flaps are used to recreate the shape and volume of the amputated breast. In some situations, the reconstruction with autologous tissue is the only valid option, e.g. when there is not enough skin envelope or when the post mastectomy skin is difficult or impossible to expand due to radiotherapy and fibrosis [**3**,**4**]; 

• breast reconstruction with autologous tissue and implant. 

For this study, we have analyzed the data from 80 patients with stage I and II breast cancer who had immediate or delayed breast reconstruction and we have looked for the factors that might influence the final outcome of each procedure. We have taken into account the patient’s characteristics (age, BMI, smoker/non smoker / tumor size at diagnosis/treatments received for breast cancer / co-morbidities/axillary sampling / axillary clearance), technical details of the procedure (mastectomy weight/flap type / flap weight / unilateral / bilateral / artery time / vein time / ischemic flap time / donor vessels / per operative complications / operating time / post operative complications / return to theater / late complications) and we have studied the relations between the items. 

## Results and discussions

The patient’s age and BMI had no influence on the complications rate (per operative, post operative or late complications.

**Fig. 1:**
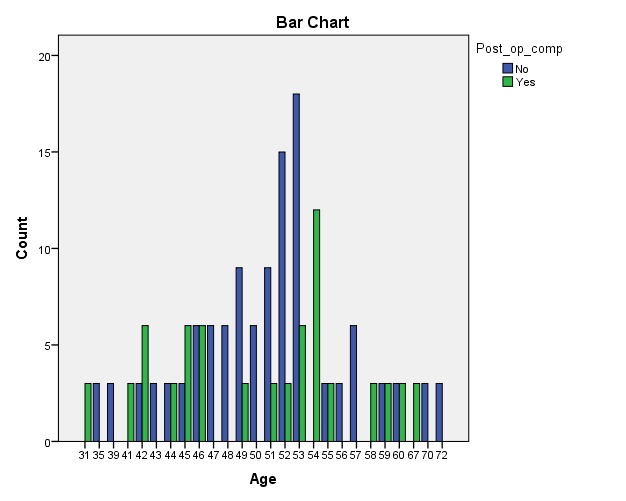
Complications rate according to age and BMI

The data shows a much higher rate of postoperative complications in smoker comparative to non-smoker patients (50%). This is not the case for the late complications. The number of late complications was small and equal for the two groups.

The operating time is well correlated with an increase number of postoperative complications. In addition, it was noted that the longer the operating time the longer the postoperative recovery.

Per operative complications are not related to the time needed for the arterial anastomosis, but a significant increase in the rate of complications was noticed to be due to a prolonged time of venous anastomosis.

**Fig. 2:**
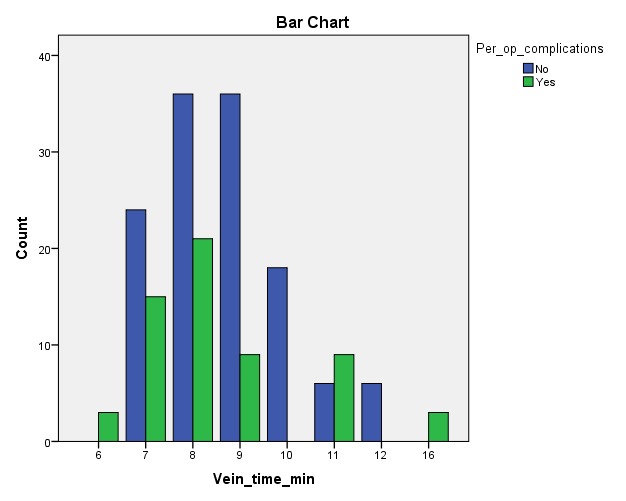
Venous anastomosis

The flap ischemic time (35-115 minutes) had no impact on the type or number of complications. 

From a surgical point of view, there was no difference between the immediate and delayed breast reconstruction regarding the postoperative complications and the recovery time.

The patients undergoing bilateral reconstruction needed a slightly longer recovery time.

## Conclusions

When performed in a multidisciplinary team to ensure the oncologic safety, breast reconstruction represents an important stage in the treatment of breast cancer. 

The rate of complications is low and it depends on the right timing of the surgery and the technique used, including the surgeon’s experience.

**Acknowledgement**

 This paper is partly supported by the Sectorial Operational Programme Human Resources Development, financed from the European Social Fund and by the Romanian Government. 
